# Prospective study of 136 adolescent scoliosis of more than 40° treated with the Lyon brace

**DOI:** 10.1186/1748-7161-8-S1-O34

**Published:** 2013-06-03

**Authors:** JC De Mauroy

**Affiliations:** 1 Clinique du Parc, Lyon, France

## Background

Due to a lack of prospective studies, which are difficult to perform, bracing adolescent scoliosis is discussed from 30°, and sometimes surgery is proposed at this angulation. It seems interesting to study the results of scoliosis over 40° with the Lyon brace, and to present the criteria for successful treatment.

## Aim

To prove the effectiveness of the Lyon conservative treatment in the most difficult conditions. If treatment is effective for scoliosis over 40°, it is even more so for lower angulations.

## Methods

We selected in our prospective database a cohort of 176 adolescent scoliosis patients with curves over 40° seen between 1998 and 2007. At this angulation, the Lyon management involves 2 plaster casts, each worn for two months, and a full time Lyon brace up to 18 months after the end of the growth, and a specific program of physiotherapy (Lyon method).

## Results

24 patients (13.6%) did not accept the protocol and chose surgery. 11 patients (6.25%) discontinued during the treatment. 136 patients (136 females and 18 males) can be studied statistically. Mean initial angulation is 45.4° (+-6.33); reducibility in plaster cast and brace is 50%. At follow up 2 years after weaning, angulation is 40.5° (+-13.1). 61 patients (45%) improved by more than 5°, 27 patients (20%) are stable, 48 (35%) are worsening by more than 5°. The results are worse in boys (57% failure). 21 patients were evaluated 10 years after weaning of the brace. Mean progression during this period is 5° (0.5°/year) identical to that of the general adult scoliotic population. The best results are obtained when the lower vertebra is L3 (89%); the worst when the lower vertebra is T12 (53%). The two significant predictors of failure of conservative treatment are:

- A prior treatment (p>0,05).

- Early onset scoliosis (EOS) and non-idiopathic scoliosis (p>0,01).

## Conclusions

Conservative treatment with Lyon brace and physiotherapy was effective in halting scoliosis progression in 65% of patients including prior treatments. The results of this study confirm that bracing is effective even after 40°. For EOS and non idiopathic, it allows waiting until end of growth for surgery.
